# Clinical photon-counting CT increases CT number precision and reduces patient size dependence compared to single- and dual-energy CT

**DOI:** 10.1093/bjr/tqaf052

**Published:** 2025-03-08

**Authors:** Jessica D Flores, Gavin Poludniowski, Adrian Szum, Georg Walther, Johan Lundberg, Patrik Nowik, Tobias Granberg

**Affiliations:** Department of Clinical Neuroscience, Karolinska Institutet, Stockholm, 141 86, Sweden; Department of Nuclear Medicine and Medical Physics, Karolinska University Hospital, Stockholm, 141 86, Sweden; Department of Nuclear Medicine and Medical Physics, Karolinska University Hospital, Stockholm, 141 86, Sweden; Department of Clinical Science, Intervention and Technology, Karolinska Institutet, Huddinge, 141 52, Sweden; Department of Clinical Neuroscience, Karolinska Institutet, Stockholm, 141 86, Sweden; Department of Neuroradiology, Karolinska University Hospital, Stockholm, 141 86, Sweden; Department of Clinical Neuroscience, Karolinska Institutet, Stockholm, 141 86, Sweden; Department of Clinical Neuroscience, Karolinska Institutet, Stockholm, 141 86, Sweden; Department of Neuroradiology, Karolinska University Hospital, Stockholm, 141 86, Sweden; Department of Clinical Science, Intervention and Technology, Karolinska Institutet, Huddinge, 141 52, Sweden; Siemens Healthineers, Solna, 169 79, Sweden; Department of Clinical Neuroscience, Karolinska Institutet, Stockholm, 141 86, Sweden; Department of Neuroradiology, Karolinska University Hospital, Stockholm, 141 86, Sweden

**Keywords:** photon-counting CT, CT number, CT number variation, precision, accuracy, beam hardening

## Abstract

**Objectives:**

To study whether photon-counting computed tomography (PCCT) can improve CT number accuracy and precision and reduce patient size dependence compared to dual-energy CT (DECT) virtual monoenergetic imaging (VMI) and single-energy CT (SECT).

**Methods:**

Clinical PCCT, DECT, and SECT scanners were used to image a multi-energy quality assurance phantom and tissue-equivalent inserts with/without an outer nested annulus, representing 2 object sizes (18 and 33 cm). CT numbers were converted to linear attenuation coefficients (LAC) and regions of interest applied. Theoretical monoenergetic LAC were calculated from known elemental compositions as a ground truth. Percent differences in mean LAC between phantom sizes, between mean and theoretical LAC, and its coefficient of variation (COV) were calculated.

**Results:**

Mean LAC percent differences between small and larger phantoms were highest in DECT (within −3% to 9%) and SECT (within 1%-5%), particularly at higher calcium and iodine concentrations, while being relatively constant in PCCT over material concentrations and VMI energies (within ±2%). The COV in mean LAC was consistently lower (about 2-5 times) in PCCT relative to DECT and SECT for calcium in the large phantom. With consideration of the theoretical uncertainties of 2%, both PCCT and DECT showed comparable agreement to theoretical LAC.

**Conclusions:**

PCCT VMI produces CT numbers with less dependence on patient size and increased precision in large object sizes than DECT VMI and SECT.

**Advances in knowledge:**

Clinical PCCT provides less variable CT numbers than DECT and SECT with less sensitivity to the imaged object size.

## Introduction

Until recently, clinical CT systems, both dual-energy (DECT) and single-energy CT (SECT), have relied on energy-integrating detectors (EIDs). In 2021, the first clinical whole-body spectral CT system with photon-counting detectors (PCD) was released. Previous photon-counting CT (PCCT) studies have shown improvements in the spatial resolution,^1^ contrast-to-noise ratio,^2^ dose efficiency,^3^^,^^4^ image noise,^2^^,^^3^ structural visualization,^5^ and quantitative imaging.^1^^,^^5^ The clinical benefits of PCCT could therefore be substantial.

Current clinical CT implementations with EIDs and PCDs rely on scintillation detectors and cadmium-telluride semiconductor detectors, respectively. Unlike EIDs, PCDs allow for the direct conversion of individual x-ray photons into electrical signal.^1^ Furthermore, PCDs can discriminate single photons based on their energies,^6^ and typically, single photons are assigned to certain energy channels. EID-based DECT is limited to only 2 channels which may overlap, while PCDs can measure with more than 2 energy channels.^7^ Additionally, EIDs add both Swank noise^8^ and electronic noise,^1^^,^^7^ and weigh lower energy photons less, resulting in reduced contrast and increased noise. PCDs, however, can reject electronic noise with modification of the lowest energy threshold for each energy channel.^7^ PCCT, therefore, has the potential to improve quantitation beyond that attainable with DECT.

Additionally, CT number variation for a given material and incident photon energy should ideally be minimal within a homogeneous region. The CT number for a specified x-ray energy should also be independent of acquisition settings.^9^ However, due to differences in x-ray source spectra, detector performance, and post-processing, CT numbers may vary between acquisition settings and CT systems.^10^ To combat this issue and to consider the energy dependence of CT numbers, virtual monoenergetic imaging (VMI) in DECT^11^ and PCCT may be used, allowing for the assignment of Hounsfield unit (HU) values to CT numbers at standard photon energies that have clear physical meaning.^1^^,^^10^ Prior investigations have shown decreased CT number variation with DECT over SECT.^12^^,^^13^ In addition, PCCT has been shown to further reduce CT number variation across a range of radiation doses compared to DECT.^3^^,^^14^ Given that SECT is widely used, it may be beneficial to extend CT number variation investigations in PCCT to both DECT and SECT.

When imaging large objects, measured CT numbers may vary in the same material. This shortcoming is relevant, for example, in radiation therapy treatment planning, in which CT numbers are used to calibrate electron density values^15^ in patients of all sizes. DECT has shown stabilized CT numbers across object sizes.^12^ Additionally, composition characterization and volume quantification of urinary stones are key parameters in treatment decisions for suspected urolithiasis and are typically performed with CT number threshold-based methods.^16^ Renal cystic masses are also defined by their attenuation and their risk of malignancy is based on an attenuation-dependent Bosniak classification system.^17^ Because CT numbers may vary and are energy dependent, a study^10^ has advocated for the adoption of a series of standard energies or VMI for tissue characterization. In applications where highly accurate and precise CT numbers are required, PCCT may therefore be clinically valuable.

Seeing the potential clinical benefits of PCCT in diagnostic imaging and therapy, the scope of this study was to characterize CT number variation in clinical PCCT compared to DECT and SECT in terms of accuracy and precision by investigating the noise caused by phantom size, material concentration, and virtual monoenergetic imaging.

## Methods

### Image acquisition and reconstruction

Imaging was performed on a first-generation clinical dual-source PCCT (NAEOTOM Alpha, VA50, Siemens Healthineers, Forchheim, Germany) in the single-source, multi-energy mode (Quantum Plus) and a second-generation clinical dual-source CT system (SOMATOM Definition Flash, VB20, Siemens Healthineers, Forchheim, Germany) in both the DECT and SECT modes. Spiral factory head and abdomen protocols were used and modified to turn off the automatic exposure control to keep the CT dose index (${\mathrm{CTDI}}_{\mathrm{vol}}$) constant and to modulate tube voltage. The phantoms were scanned with abdominal protocols using a ${\mathrm{CTDI}}_{\mathrm{vol}}$ setting of 10 mGy and 20 mGy with PCCT, DECT and SECT. The tube potential was 140 kVp in PCCT and SECT, while the tube potential pair was 140 kVp and 80 kVp without a tin filter in DECT. The SECT tube potential was chosen so that it resulted in an x-ray spectrum with a mean energy closest to 70 keV in order to more fairly compare SECT clinical protocols with 70 keV VMI PCCT and DECT. Images were reconstructed with Qr40 and Br40 kernels. Standard iterative reconstruction strength settings in the factory protocols were used: advanced modelled iterative reconstruction strength of 3 for DECT and SECT, and quantum iterative reconstruction strength of 3 for PCCT. Images were reconstructed with 5.0 mm slice thickness and 5.0 mm slice increment. PCCT and DECT images were reconstructed at various virtual monoenergetic image (VMI) energy levels: 40, 60, 70, 90, 110, and 130 keV. Both phantom sizes were scanned with the same protocols. Every protocol/phantom size configuration scan was repeated 5 times to account for statistical variation in the reconstructed images. Table 1 summarizes the image acquisitions and reconstructions in this work.

**Table 1.** Image acquisition and reconstruction configurations.

Scanner	Protocol	Tube voltage (kVp)	Effective current time product (mAs)	Rotation time (s)	CTDIvol (mGy)	Pitch	Acquisition (mm)	Kernel
PCCT	Abdomen Factory	140	87	0.5	10	0.8	144 × 0.40	Qr40, Br40
	Brain Factory		72		20	0.35	48 × 0.40	Qr40
SECT	Abdomen Routine IR	140	101	0.5	10	0.6	128 × 0.60	Qr40, Br40
	Head Neuro IR		71	1.0	20	0.55	40 × 0.60	Qr40
DECT	DE Abdomen VNC IR	140/80	37/204	0.5	10	0.6	32 × 0.6	Qr40, Br40
	DE Head Brain Hem IR		39/166		20	0.7	40 × 0.60	Qr40

All protocols are factory protocols. CTDIvol = CT dose index; DE and DECT = dual-energy CT; IR = iterative reconstruction; PCCT = photon-counting CT; SECT = single-energy CT; VNC = virtual non-contrast.

### Phantom

A multi-energy quality assurance phantom (Model 662, Computerized Imaging Reference Systems [CIRS], Inc., Norfolk, Virginia, USA) was imaged both with and without the outer nested plastic water disk, representing a small (18 cm) and a large (33 cm) object. The small object represents a head configuration while the large object represents an abdomen configuration. Such object sizes corresponded closely to the physical sizes of phantoms which are typically used in quality control (ie, ${\mathrm{CTDI}}_{\mathrm{vol}}$ measurement) tests. Removable tissue equivalent material inserts were placed in the non-central parts of the inner plastic water disk of the phantom. Inserts included a series of iodine in blood (0.0, 0.5, 2.0, 5.0, 10.0, 15.0 mg/mL) and calcium in water (10, 20, 40, 60, 120, 240 mg/mL).

**Figure 1.**
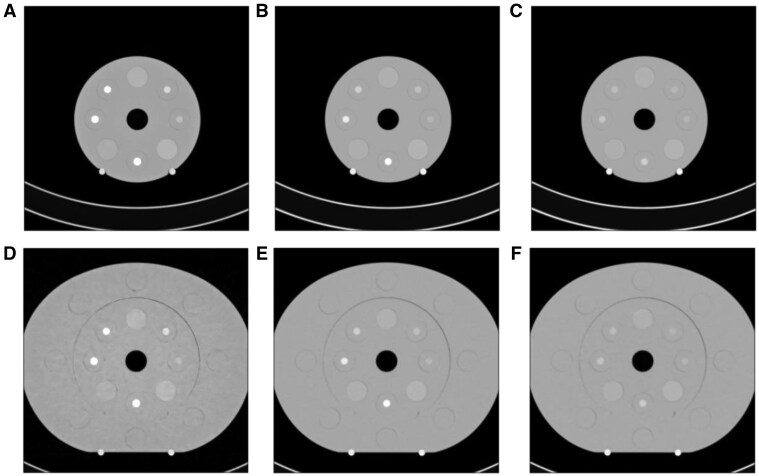
Axial PCCT images of the (A-C) small and (D-F) large phantom configurations. Reconstructions were made with a Qr40 kernel and VMI. (A, D) 40 keV, (B, E) 70 keV, and (C, F) 110 keV VMI images are shown. Starting from the 12 o’clock position and moving in the clockwise direction, the material inserts that are shown include brain grey matter, 2 mg/mL of iodine in blood, 0.5 mg/mL of iodine in blood, blood, 15 mg/mL of iodine in blood, brain white matter, 10 mg/mL of iodine in blood, and 5 mg/mL of iodine in blood. The window level is -300 HU and the window width is 1400 HU. PCCT = photon-counting CT; VMI = virtual monoenergetic imaging; HU = Hounsfield units.

To ensure that the phantom did not lay in a rotated position, the mattress on the table was removed. To ensure a true isocentric position, the phantom’s superficial markings were aligned with the CT system’s lasers. Additionally, the edges of the nested disk were made flush when creating the large phantom and the overall phantom edge (for both phantom sizes) was made parallel to the laser in the lateral direction. Finally, the phantom was not repositioned between the scans. Examples of the phantom set-up and imaging acquisitions are shown in Figure 1.

### Image analysis

The images from each scan were loaded and analyzed using Python 3 (Python Software Foundation) and the Pydicom library.^18^ A custom-made script allowed for manual placement of circular regions of interest (ROI) with a radius of 3 voxels on each material insert for 4 central slices of each scan, ensuring that ROIs were not placed near insert edges in the axial plane. Given a phantom size and scanner, the same ROIs were used for all scans. Some inserts, like calcium, had material cores, and for this reason, a larger ROI was impractical. CT numbers in Hounsfield units were converted to linear attenuation coefficients (LAC) in cm^−1^ units to provide a ratio variable with a non-arbitrary zero such that a zero value corresponds to no attenuation.^19^ Because some of the quantitative metrics used in this work rely on ratios, this unit conversion also ensured that the denominator was non-zero in the ratios of interest. According to the definition of the Hounsfield unit,^10^ CT numbers were converted to LAC as, $\mathrm{LAC}=\left(\mathrm{CT}\;\mathrm{number}+1000\right)\times {\mathrm{LAC}}_{\mathrm{water}}/1000,$ where ${\mathrm{LAC}}_{\mathrm{water}}$ is the theoretical LAC of water. The calculation of theoretical LAC values is further described in the following section. The mean and standard deviation (SD) across 4 central slices and 5 repeated scans were calculated within the ROIs. Thus, 20 individual mean and standard deviation data points per protocol, phantom size, and material were acquired. Background, within-ROI SD in mean LAC (ie, the “noise”) was calculated as the mean SD within these ROIs for blood or 0 mg/mL of iodine in blood.

### CT number accuracy and precision

Measured LAC were compared to calculated theoretical ground truth values. Theoretical LAC were calculated using batch-specific elemental compositions and densities of tissue equivalent material inserts, which were supplied by the manufacturer. The SpekPy software toolkit V2^20^^,^^21^ was used to calculate total LAC in Python, using the default renormalized cross-sections from the PENELOPE Monte Carlo code system.^22^ Because SECT does not yield unique CT numbers for a given material and tube potential, it was not possible to calculate theoretical LAC for SECT while maintaining generality (ie, assuming no dependence on the object), and without making assumptions regarding the beam quality and detector response. For these reasons, only a comparison of LAC accuracy in PCCT and DECT VMI is relevant. Overall accuracy was assessed by calculating the percent difference between mean measured and theoretical LAC and is reported with percentage units; this was calculated as the difference between mean measured and theoretical LAC divided by theoretical LAC. Overall precision was assessed by calculating the coefficient of variation (COV) of the mean measured LAC for PCCT, DECT, and SECT; this was calculated as the standard deviation of the mean measured LAC divided by the overall mean LAC.

### CT number differences due to phantom size

The impact of beam hardening was assessed by comparing large and small phantom LAC. Percent differences are reported with percentage units and were calculated as the difference between large and small phantom LAC divided by the average of large and small phantom LAC.

### Statistical analysis

Metrics were calculated for every VMI energy (if applicable in PCCT and DECT), material, concentration, and phantom size for each scanning mode (ie, PCCT, DECT, and SECT). 95% confidence intervals (CI) were calculated using percentile bootstrap sampling with datasets randomly sampled 1000 times. All error bars in the plots represent 95% CI of metrics.

A detailed description of the metrics used in this study and results for within ROI SD in PCCT, DECT, and SECT are available in the Supplemental Material Section S1.

## Results

Results are presented here for a single dose level and reconstruction kernel (a ${\mathrm{CTDI}}_{\mathrm{vol}}$ of 10 mGy and the Qr40 kernel). A similar behaviour was observed for the alternate dose level (20 mGy) and reconstruction kernel (Br40), over various material concentrations in PCCT, DECT, and SECT as well as VMI energies in PCCT and DECT.

### Calcium concentration: comparison of SECT to 70 keV VMI of PCCT and DECT

**Figure 2.**
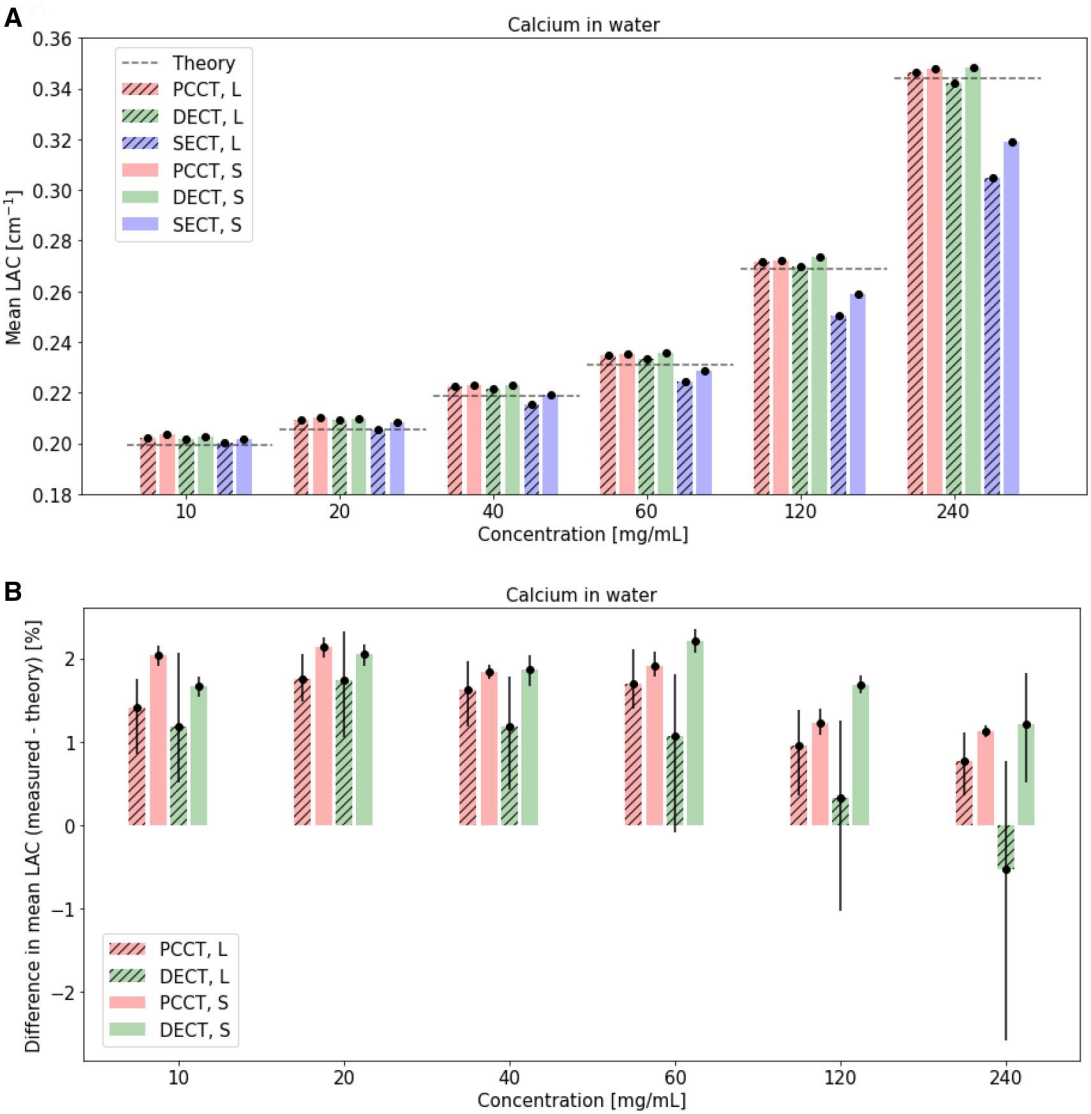
(A) Mean LAC and (B) percent difference between measured mean and theoretical LAC in a small and large phantom over a range of calcium concentrations in PCCT, DECT, and SECT. 95% confidence intervals are shown as error bars and dotted lines in indicate theoretical LAC. PCCT and DECT virtual monoenergetic images at 70 keV are shown. PCCT = photon-counting CT; DECT = dual-energy CT; SECT = single-energy CT; S = small phantom; L = large phantom; LAC = linear attenuation coefficients.

**Figure 3.**
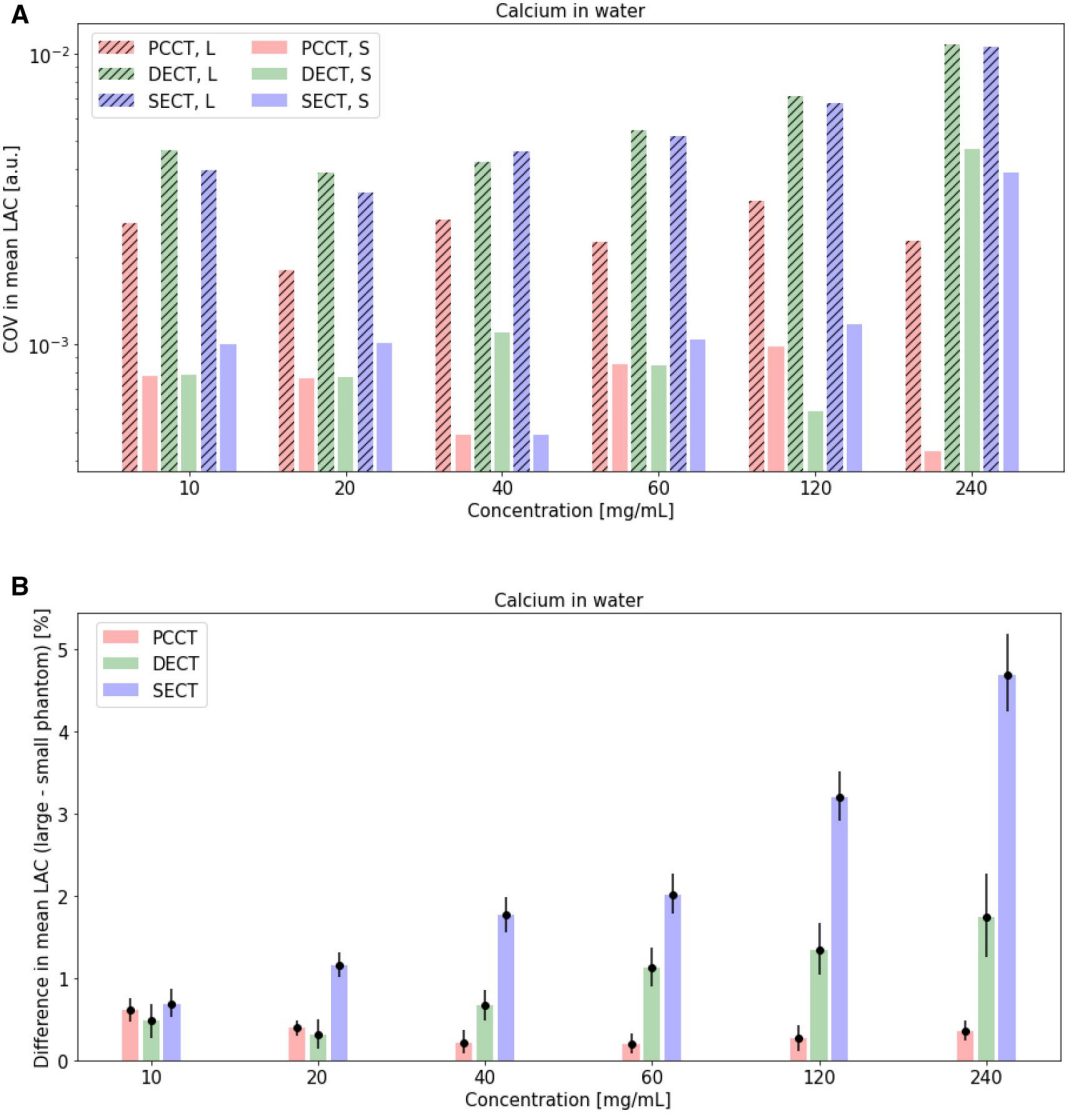
(A) COV in measured mean in a small and large phantom over a range of calcium concentrations in PCCT and DECT, and (B) percent difference in mean LAC of large and small phantoms over a range of calcium concentrations in PCCT, DECT, and SECT. 95% confidence intervals are shown as error bars. PCCT and DECT virtual monoenergetic images at 70 keV are shown. PCCT = photon-counting CT; DECT = dual-energy CT; SECT = single-energy CT; S = small phantom; L = large phantom; LAC = linear attenuation coefficients; COV = coefficient of variation; a.u. = arbitrary units.

Mean LAC (Figure 2A), percent difference between measured mean and theoretical LAC (Figure 2B), COV in measured mean LAC (Figure 3A), and percent difference in mean LAC of the large and small phantom (Figure3B) for various calcium insert concentrations in small and large phantoms are shown. For all calcium concentrations, mean LAC measurements in both small and large phantoms were near theoretical LAC values in PCCT and DECT (Figure 2A), as also indicated by low percent differences between measured the mean and theoretical LAC (Figure 2B). For both PCCT and DECT, percent differences between the mean and theoretical LAC were within about 2% (Figure 2B).

PCCT data showed lower COV in mean LAC (about >1 to 5 times lower) than DECT and SECT data in the large phantom for all calcium concentrations (Figure 3A). COV values remained relatively constant for both the large and small phantom in PCCT and increased for the large phantom in DECT and SECT. Mean LAC percent differences between small and larger phantoms were highest with the higher calcium concentrations in DECT and SECT, increased with calcium concentration in DECT and SECT (Figure 3B) and remained relatively constant in PCCT.

### Iodine concentration: comparison of SECT to 70 keV VMI of PCCT and DECT

**Figure 4.**
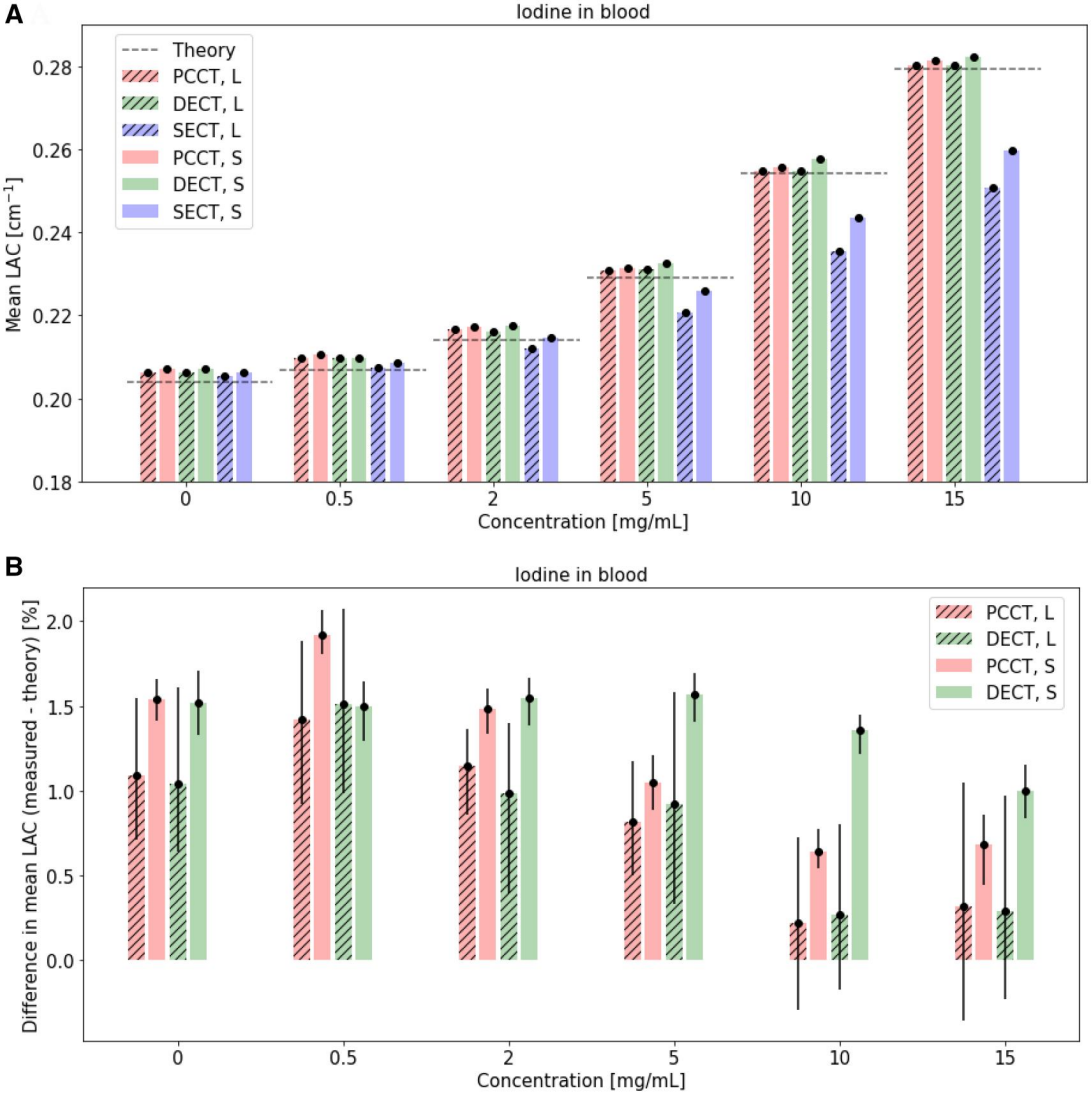
(A) Mean LAC and (B) percent difference between measured and theoretical LAC in a small and large phantom over a range of iodine concentrations in PCCT, DECT, and SECT. 95% confidence intervals are shown as error bars and dotted lines in indicate theoretical LAC. PCCT and DECT virtual monoenergetic images at 70 keV are shown. PCCT = photon-counting CT; DECT = dual-energy CT; SECT = single-energy CT; S = small phantom; L = large phantom; LAC = linear attenuation coefficients.

**Figure 5.**
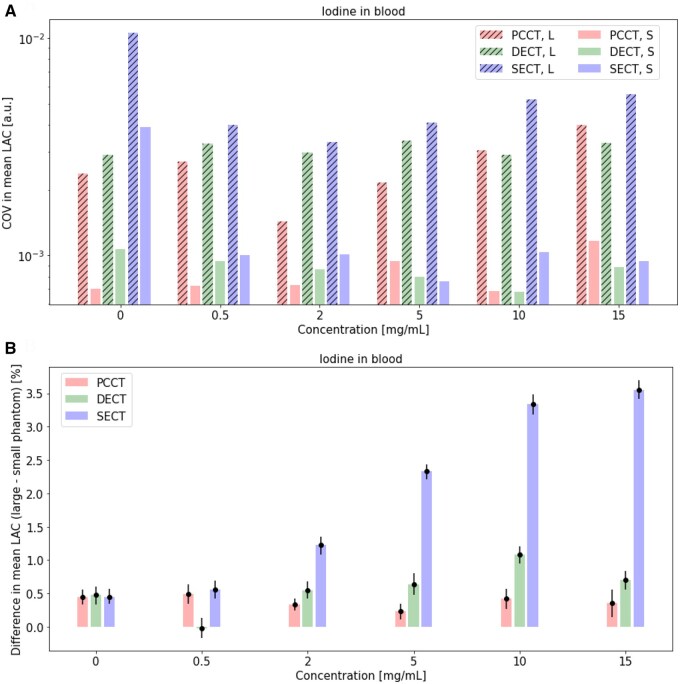
(A) COV in measured mean LAC in a small and large phantom over a range of iodine concentrations in PCCT and DECT, and (B) percent difference in mean LAC of the large and small phantoms over a range of iodine concentrations in PCCT, DECT, and SECT. 95% confidence intervals are shown as error bars. PCCT and DECT virtual monoenergetic images at 70 keV are shown. PCCT = photon-counting CT; DECT = dual-energy CT; SECT = single energy CT; S = small phantom; L = large phantom; LAC = linear attenuation coefficients; COV = coefficient of variation; a.u. = arbitrary units.

Mean LAC (Figure 4A), percent difference between measured mean and theoretical LAC (Figure 4B), COV in measured mean LAC (Figure 5A) and percent difference in mean LAC of the large and small phantom (Figure 5B) for various iodine insert concentrations in blood in small and large phantoms are shown. Background noise from the blood insert (0 mg/mL iodine) is tabulated in Table 2 and can be found in Figure S2.

**Table 2.** SD of mean background LAC in PCCT, DECT, and SECT.

Phantom size	PCCT SD ($\times 10^{-3}$cm^-1^)	DECT SD ($\times 10^{-3}$cm^-1^)	SECT SD ($\times 10^{-3}$cm^-1^)
Large	1.30 [1.20, 1.41]	2.00 [1.80, 2.20]	1.88 [1.72, 2.04]
Small	0.47 [0.44, 0.50]	0.69 [0.64, 0.74]	0.68 [0.60, 0.77]

95% confidence intervals of background (ie, blood or 0 mg/mL of iodine in blood), within ROI SD in mean LAC in PCCT, DECT, and SECT. 70 keV VMI images in PCCT and DECT are tabulated. PCCT = photon-counting CT; DECT = dual-energy CT; SECT = single-energy CT; SD = standard deviation; LAC = linear attenuation coefficients; VMI = virtual monoenergetic image.

For all iodine concentrations, mean LAC measurements in both small and large phantoms were close to theoretical LAC values in PCCT and DECT (Figure 4A). For both PCCT and DECT, percent differences between the mean and theoretical LAC were within about 2% (Figure 4B). PCCT and DECT data showed comparable COV in mean LAC for all iodine concentrations in the small and large phantoms (Figure 5A). PCCT, DECT, and SECT showed comparable COV in mean LAC in non-zero iodine concentrations in the small phantom. Additionally, percent differences between small and larger phantom mean LAC increased with higher iodine concentrations in SECT (Figure 5B). For iodine concentrations of 2 mg/mL and greater, PCCT showed lower percent differences than DECT and SECT, while DECT showed lower percent differences than SECT (Figure 5B). Furthermore, PCCT showed lower within-ROI SD in mean LAC of the background (approximately 30% lower noise) for both phantom sizes (Table 2).

### Calcium concentration: effect of VMI energy in PCCT and DECT

**Figure 6.**
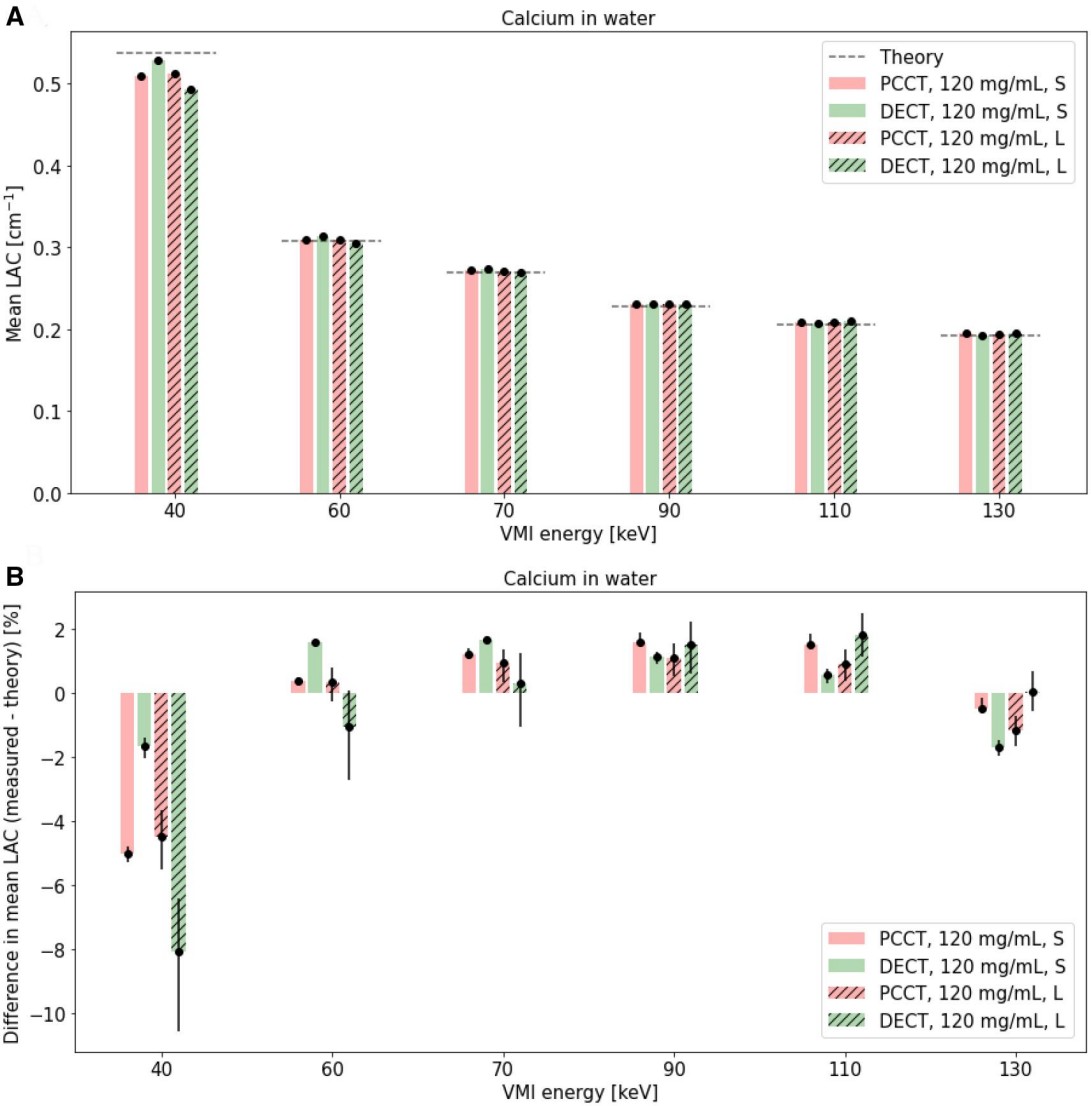
(A) Mean LAC and (B) percent difference between measured mean and theoretical LAC in a small and large phantom over a range of VMI energies in PCCT and DECT VMI. 95% confidence intervals are shown as error bars and dotted lines in indicate theoretical CT numbers. PCCT = photon-counting CT; DECT = dual-energy CT; S = small phantom; L = large phantom; LAC = linear attenuation coefficients; VMI = virtual monoenergetic imaging.

**Figure 7.**
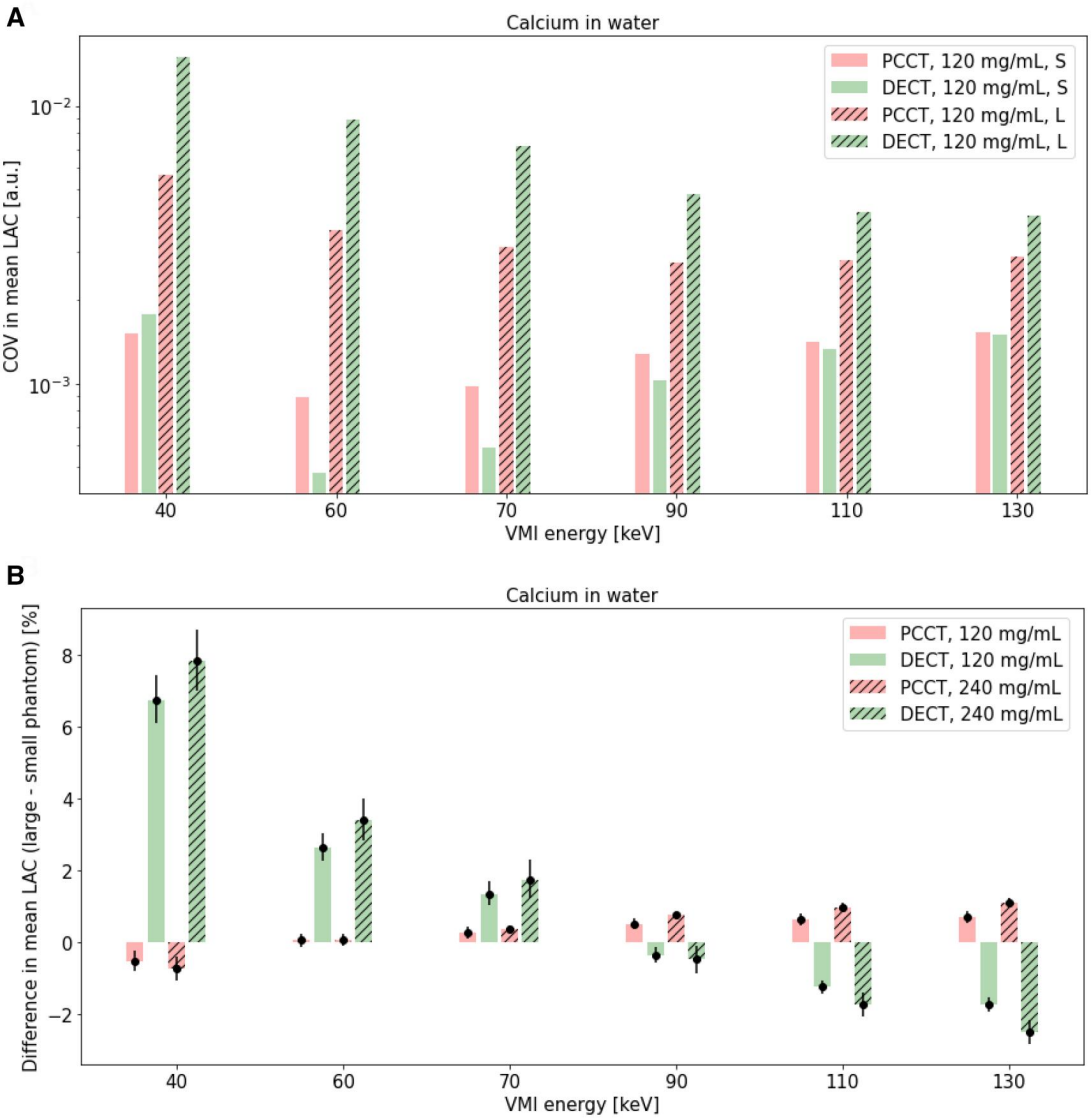
(A) COV in measured mean LAC in a small and large phantom and (B) percent difference in mean LAC of the large and small phantoms over a range of VMI energies in PCCT and DECT VMI. 95% confidence intervals are shown as error bars. PCCT = photon-counting CT; DECT = dual-energy CT; S = small phantom; L = large phantom; LAC = linear attenuation coefficients; COV = coefficient of variation; a.u. = arbitrary units; VMI = virtual monoenergetic imaging.

Mean LAC (Figure 6A), percent difference between measured mean and theoretical LAC (Figure 6B), COV in measured mean LAC (Figure 7A), and percent difference in mean LAC of the large and small phantom (Figure 7B) for calcium in small and large phantoms over various VMI energies are shown.

For the lowest VMI energy (ie 40 keV), larger deviations from theoretical LAC values (Figure 6A) and higher percent difference between measured mean and theoretical LAC values were seen in both PCCT and DECT (Figure 6B). In PCCT and DECT, percent differences between the mean and theoretical LAC were comparable in the large phantom and fell within about 2% for VMI energies greater than 40 keV (Figure 6B). COV in mean LAC was comparable for PCCT and DECT in the small phantom but largest for DECT in the large phantom (Figure 7A). Additionally, percent difference between large and small phantom mean LAC increased away from a VMI energy of 70 keV in DECT more than in PCCT (Figure 7B). For all VMI energies except 90 keV, the largest percent difference between phantom sizes was seen in DECT.

### Iodine concentration: effect VMI energy in PCCT and DECT

**Figure 8.**
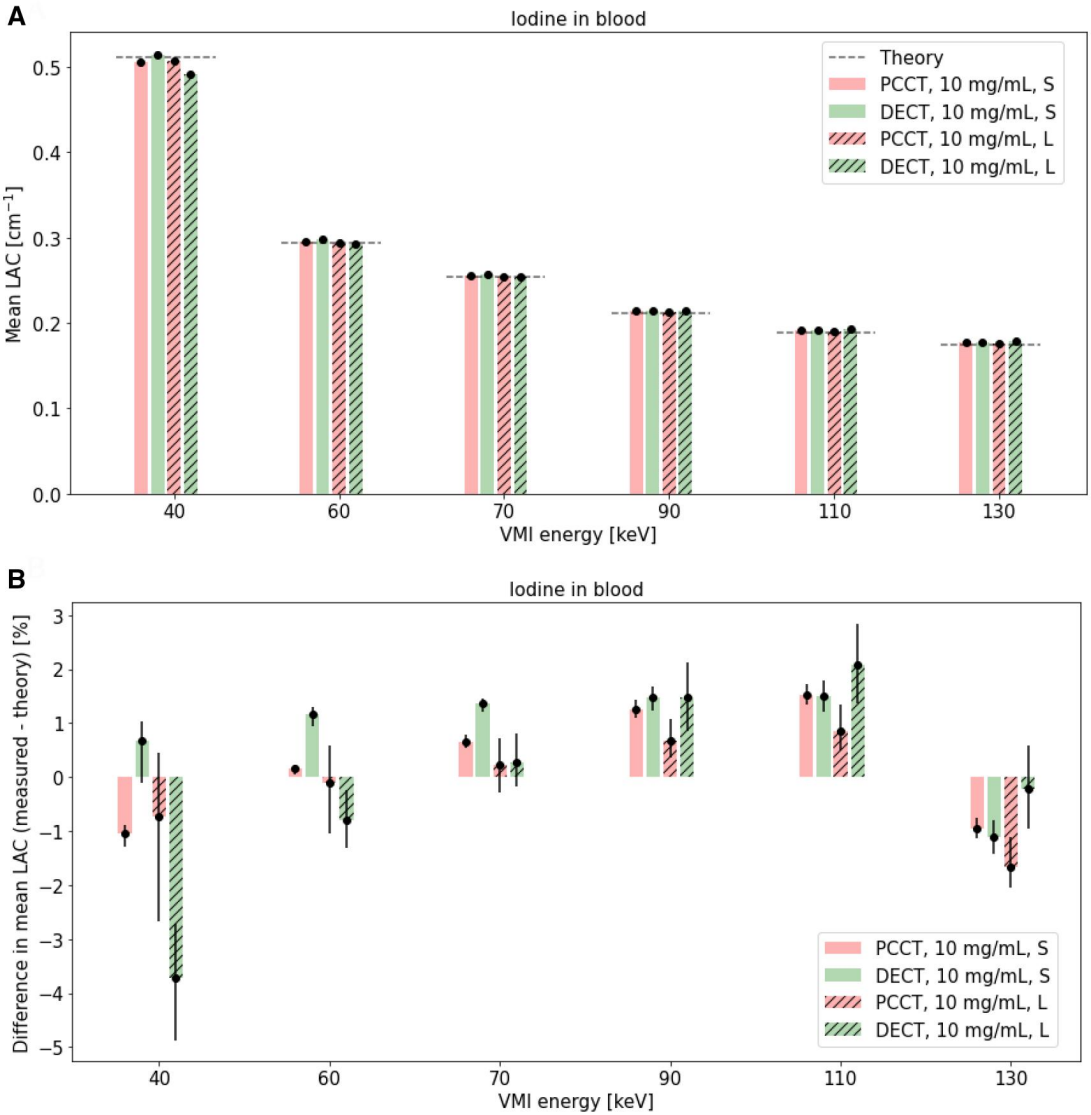
(A) Mean LAC and (B) percent difference between measured mean and theoretical LAC in a small and large phantom over a range of VMI energies in PCCT and DECT VMI. 95% confidence intervals are shown as error bars and dotted lines in indicate theoretical CT numbers. PCCT = photon-counting CT; DECT = dual-energy CT; S = small phantom; L = large phantom; LAC = linear attenuation coefficients; VMI = virtual monoenergetic imaging.

**Figure 9.**
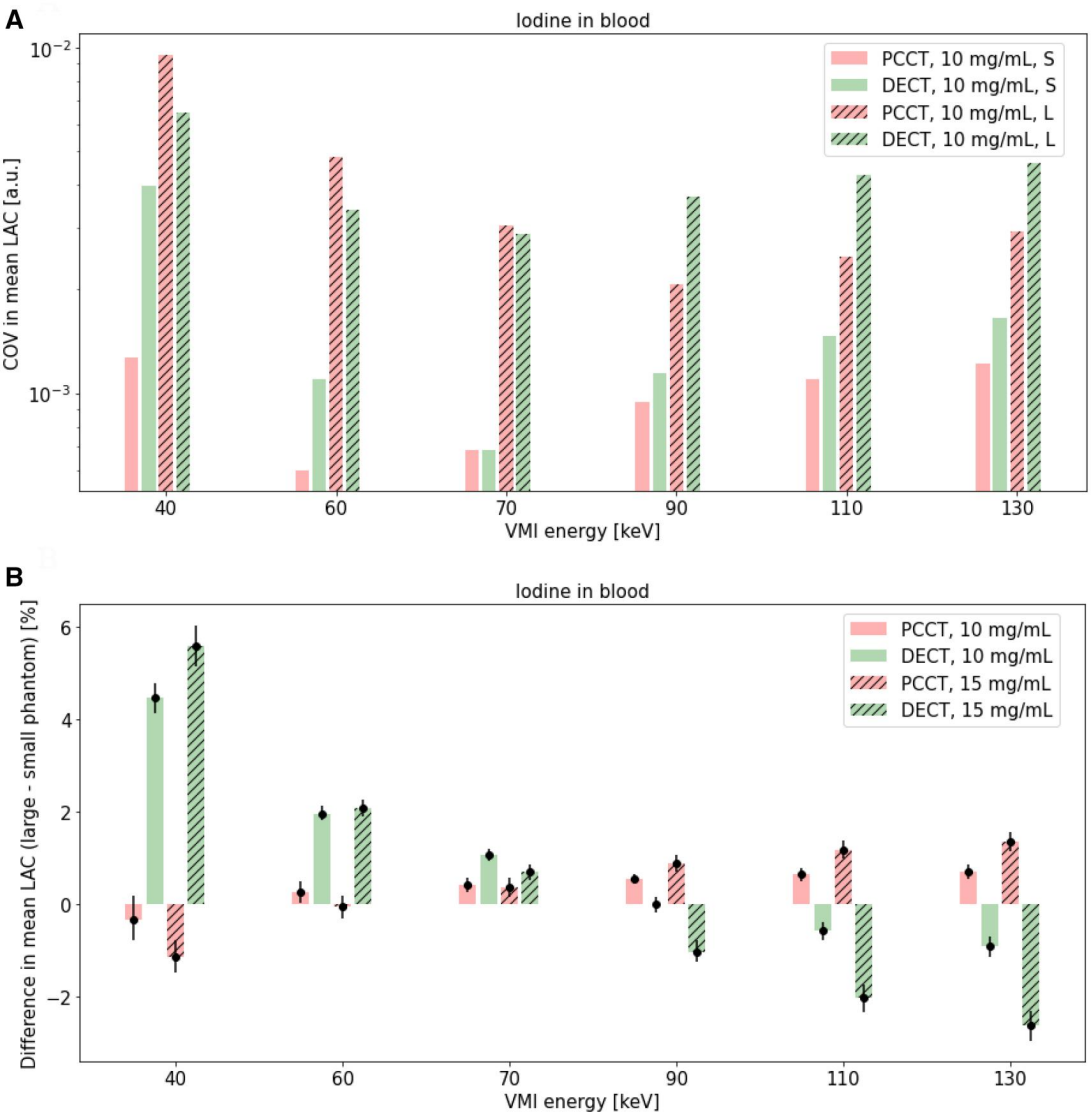
(A) COV in measured mean LAC in a small and large phantom and (B) percent difference in mean LAC of the large and small phantoms over a range of VMI energies in PCCT and DECT VMI. 95% confidence intervals are shown as error bars. PCCT = photon-counting CT; DECT = dual-energy CT; S = small phantom; L = large phantom; LAC = linear attenuation coefficients; COV = coefficient of variation; a.u. = arbitrary units; VMI = virtual monoenergetic imaging.

Mean LAC (Figure 8A), percent difference between measured mean and theoretical LAC (Figure 8B), COV in measured mean LAC (Figure 9A), and percent difference in mean LAC of the large and small phantom (Figure 9B) for iodine in small and large phantoms over various VMI energies are shown.

Percent difference between measured mean and theoretical LAC values were generally comparable in PCCT and DECT (Figure 8A and B). Overall, PCCT data showed slightly lower COV in mean LAC than DECT over all VMI energies in the small phantom and at VMI energies greater than 70 keV in the large phantom (Figure 9A). Percent difference in mean LAC between the large and small phantom increased more in DECT than PCCT as the VMI energy was varied from 70 keV (Figure 9B). For all VMI energies except 90- and 110 keV, the percent difference deviation between phantom sizes was highest in DECT, particularly at the highest iodine concentration (Figure 9B).

Table 3 summarizes the accuracy, precision and size dependence of LAC in PCCT, DECT, and SECT for 120 mg/mL of calcium in water and 5 mg/mL of iodine in blood.

**Table 3.** Accuracy, precision, and size dependence of LAC in PCCT, DECT, and SECT.

Material	Phantom size	PCCT	DECT	SECT
Accuracy: Difference in mean LAC (measured—theory) [%]				
Calcium	Large	0.95 [0.35, 1.37]	0.33 [-1.03, 1.24]	–
	Small	1.22 [1.08, 1.40]	1.68 [1.58, 1.79]	–
Iodine	Large	0.82 [0.50, 1.17]	0.92 [0.33, 1.58]	–
	Small	1.05 [0.89, 1.21]	1.57 [1.41, 1.69]	–
Precision: COV in mean LAC [$\times 10^{-3}$ a.u.]				
Calcium	Large	3.11	7.16	6.77
	Small	0.98	0.59	1.18
Iodine	Large	2.17	3.38	4.10
	Small	0.94	0.80	0.76
Size dependence: Difference in mean LAC (large-small phantom) [%]				
Calcium	–	0.26 [0.10, 0.44]	1.33 [1.04, 1.67]	3.21 [2.93, 3.53]
Iodine	–	0.23 [0.11, 0.34]	0.64 [0.48, 0.79]	2.33 [2.22, 2.44]

95% confidence intervals of accuracy and size dependence metrics in PCCT, DECT, and SECT are shown in brackets. Values for 120 mg/mL of calcium in water and 5 mg/mL of iodine in blood are shown. 70 keV VMI in PCCT and DECT are tabulated. Accuracy in SECT could not be determined. PCCT = photon-counting CT; DECT = dual-energy CT; SECT = single-energy CT; LAC = linear attenuation coefficients; COV = coefficient of variance; VMI = virtual monoenergetic image.

## Discussion

With the introduction of photon-counting CT in clinical practice, there is great promise for increased CT number precision and decreased patient size dependence in CT imaging, which may allow for improved quantitative intra- and inter-subject comparisons. However, evidence of PCCT’s advantages in spectral imaging in terms of accuracy and precision is needed. To our knowledge, this study is the first to evaluate accuracy, precision, and patient size dependence of CT numbers in PCCT, DECT, and SECT for a range of material concentrations and VMI energies in the same study. Our findings show that PCCT VMI of highly attenuating materials can produce more consistent values across varying phantom size than DECT VMI and SECT, slightly improved precision in large phantom sizes, and comparable accuracy across various material concentrations and VMI energies to those in DECT. We note that this conclusion regarding precision was based on the variability of mean values in ROIs and not simply related to the lower pixel noise associated with PCCT with respect to DECT or SECT for a fixed radiation dose.

These findings may positively impact applications such as in quantitative imaging and in large patient imaging. Notably, increasing object size has been shown to exacerbate the prevalence of CT number variation, particularly in SECT.^12^ DECT VMI has been shown to reduce CT number variation across patient sizes,^12^ in addition to showing beam hardening artefact reduction.^23^ This work shows there is a further improvement in the consistency of CT numbers across object size in PCCT compared to both DECT and SECT. This suggests that there may be more reliable quantitative information on imaged anatomy in all patients, regardless of patient size and VMI energy.

The improved consistency in CT numbers using PCCT VMI could also have applications in radiation therapy. Treatment planning for radiation therapy is usually based on x-ray CT data, where CT numbers from phantom calibrations are used to calculate values of interest, such as electron density.^15^ Although CT numbers may vary with size, calibrations are often performed on a single phantom and applied to patients of all sizes, which may not work well for patients that are smaller or larger than the calibration phantom.^24^ CT number variation in PCCT may also be particularly important in proton therapy treatment planning, where CT numbers are converted to stopping power ratios via a calibration curve.^15^ Another application includes heavy ion therapy, in which concentrations of oxygen and carbon in human tissues can be derived from CT images for treatment planning.^25^ DECT has previously shown improvements in calculated values that are derived from CT numbers in both ion^25^ and proton therapy.^26^ Our results, however, demonstrate even further improvements in CT number quantitation in clinical PCCT VMI compared to DECT VMI, highlighting the opportunity for improved quantitative imaging over a range of VMI energies.

Diagnostic imaging that employs CT number analysis may also improve with implementation of PCCT in the clinic. In treatment decisions for urolithiasis, urinary stones are typically characterized using CT number threshold-based methods.^16^ Other studies have shown that DECT can accurately characterize urinary stones^27^ and that a prototype PCCT system can distinguish the composition of various urinary stone types.^28^ In the assessment of malignancy risk, renal cystic lesions are classified using a Bosniak classification system that is based on CT numbers.^17^ Some studies have shown that there is improved characterization of renal masses with DECT^29^ and that VMI on a clinical PCCT system can be used to classify renal cysts.^30^ Furthermore, CT numbers are also used to determine the proportion of tissue component in order to predict tumour invasiveness.^31^ One study has shown that DECT VMI can be useful for differentiating subtypes of solid and invasive lung lesions.^32^ Because the scanning technique impacts the CT number variation, the appropriate consideration of energy dependency differences between materials may be necessary and it may be beneficial to use VMI and a series of standard VMI energies,^10^ both in DECT and PCCT, in certain classification schemes. The adoption of a series of standard energies for tissue characterization may positively impact the management of treatment decisions that rely on threshold-based methods. Both DECT and PCCT would allow for this standardization. In diagnostic applications that rely on CT number analysis, PCCT may therefore be clinically valuable and may offer a characterization with higher precision and less size dependence than DECT.

To our knowledge, 2 groups, Liu et al^3^ and Vrbaski et al,^14^ have investigated CT number precision and have performed phantom evaluations for PCCT and DECT. Overall, there is good agreement between our findings on increased precision and reduced noise in PCCT VMI, relative to DECT VMI. However, Liu et al^3^ did not investigate CT number accuracy. Vrbaski et al^14^ also investigated CT number accuracy in 3 VMI energy levels and focused on assessing quantitative performance of PCCT in pelvic protocols with only one reconstruction kernel and a different ADMIRE strength setting. Another group, Michalak et al,^13^ has investigated CT number variation (pseudo-accuracy in terms of CT number deviation from a visually stable acquisition setting in DECT) in DECT and SECT. There is overall good agreement with these results, showing that DECT VMI can produce more consistent CT numbers than SECT. Unlike other CT number precision studies in PCCT,^3^^,^^14^ our study extends these investigations to SECT to quantify CT number accuracy and precision in PCCT VMI, DECT VMI, and SECT in abdominal protocols and assesses accuracy, over 6 VMI energy levels and 6 calcium and iodine insert concentrations, based on calculation of theoretical CT numbers from known elemental compositions of phantom inserts.

Furthermore, this study assesses accuracy with comparison of measurements to theoretical CT numbers or linear attenuation coefficients (LACs). Unlike Vrbaski et al,^14^ theoretical mass attenuation coefficients (MAC) were directly calculated from tabulated cross-sections. Such tabulations are expected to have 1%-2% error in the kilovoltage range^33^ that is relevant to CT. Additionally, there is uncertainty in the phantom insert density, which is used to calculate LAC from MAC. As a result, we expect that there is an envelope of 2% uncertainty in theoretical LAC calculations. Our results show that PCCT and DECT have comparable accuracy and that percent difference between mean measured and theoretical LAC in PCCT and DECT fall within expected levels of theoretical LAC uncertainty.

This study has strengths and limitations. Strengths include the direct comparison of PCCT, DECT, and SECT in the same experiments, the use of several different inserts (reflecting clinically relevant calcium, iodine and blood concentrations), and 2 object sizes. Limitations include that only images of a uniform, solid phantom were evaluated, which may not reflect true performance in realistic clinical tasks that are typically more complex and textured (ie, not uniform). A second limitation is that although patients come in a variety of sizes, only 2 phantom sizes were investigated in this work but the phantom used corresponded closely to physical sizes which are typically used in quality control (ie, ${\mathrm{CTDI}}_{\mathrm{vol}}$ measurement) tests. A third limitation is that although there is only one clinically available PCCT system, there are many clinical DECT and SECT systems in the market; thus, our results may not be generalizable to other models on the market. Furthermore, different implementations of DECT (dual layer, dual source, and kVp switching) are also available and have demonstrated specific noise characteristics.^34^ Because large differences were not expected between imaging systems, this study only focused on one, older generation DECT and SECT system.

## Conclusions

In conclusion, this study reports on phantom evaluations of a clinical first-generation dual-source photon-counting CT scanner with a focus on CT number accuracy, precision, and object size dependence of CT numbers over a variety of materials, material concentrations, and virtual monoenergetic imaging energies. Clinical PCCT produces CT numbers that are less dependent on phantom size and more precise in larger object sizes than both DECT and SECT.

## Supplementary Material

tqaf052_Supplementary_Data
