# Supplementary material for: Clinical photon-counting CT increases CT number precision and reduces patient size dependence compared to single- and dual-energy CT
Source: Br J Radiol. 2025 Mar 8;98(1169):721–33. doi: 10.1093/bjr/tqaf052 (PMC12012351; doi:10.1093/bjr/tqaf052)
Supplement: tqaf052_Supplementary_Data [file tqaf052_supplementary_data.zip › tqaf052_Supplementary_Data/supplementalMaterial_figure2.pdf]

Supplementary figure 2. Within ROI standard deviation in iodine in blood.

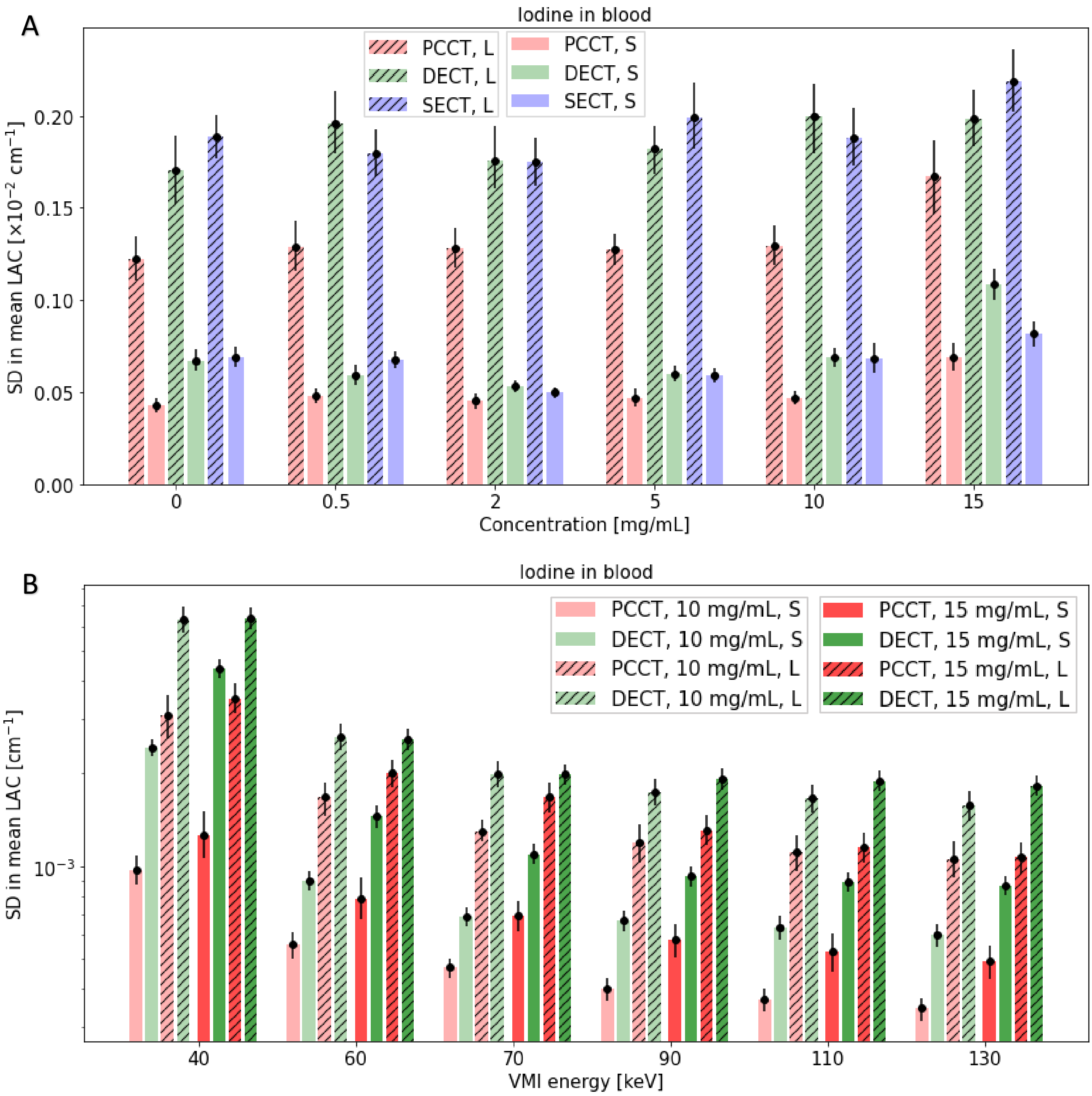

95 % confidence intervals of mean within ROI standard deviation for iodine in blood over (A) various concentrations in PCCT, DECT and SECT, and (B) VMI energies in PCCT and DECT. Confidence intervals are shown as error bars. PCCT and DECT VMI images at 70 keV are shown in A. PCCT refers to photon-counting CT; DECT refers to dual-energy CT; SECT refers to single-energy CT; S refers to the small phantom; L refers to large phantom; SD refers to standard deviation; LAC refers to linear attenuation coefficient; VMI refers to virtual monoenergetic imaging.
