# Supplementary material for: Clinical photon-counting CT increases CT number precision and reduces patient size dependence compared to single- and dual-energy CT
Source: Br J Radiol. 2025 Mar 8;98(1169):721–33. doi: 10.1093/bjr/tqaf052 (PMC12012351; doi:10.1093/bjr/tqaf052)
Supplement: tqaf052_Supplementary_Data [file tqaf052_supplementary_data.zip › tqaf052_Supplementary_Data/supplementalMaterial_text.docx]

**Supplemental Materials**

**Metrics definitions**

Within the $i^{\mathrm{th}}$ region of interest (ROI), the mean linear attenuation coefficient (LAC) is $\bar{\mu}^{i}$ and the standard deviation is $\sigma_{\mu}^{i}$. The mean LAC over all ROIs or the overall mean LAC is defined as,

$$\mu=\mathrm{mean}\left( \bar{\mu} \right)=\frac{1}{N}\sum_{i=1}^{N} \bar{\mu}^{i}$$

where $N$ is the number of ROIs (i.e. $N=20$).

Overall accuracy is assessed between ROIs as the percent difference in mean measured and theoretical LAC and is calculated as

$$\mathrm{mean}\left( \frac{\left( \bar{\mu}-\mu_{th} \right)}{\mu_{th}} \right)=\frac{1}{N}\sum_{i=1}^{N} \frac{(\bar{\mu}^{i}-\mu_{th})}{\mu_{th}},$$

where $\mu_{th}$ is the theoretical LAC. Overall precision is assessed between ROIs as the coefficient of variation (COV) in mean measured LAC and is calculated as

$$\frac{\mathrm{SD}(\bar{\mu})}{\mu}.$$

Percent difference is a measure of accuracy between ROIs for one object size with respect to the other size and is defined as,

$$\mathrm{mean}\left( \frac{2\left( \bar{\mu}_{L}-\bar{\mu}_{S} \right)}{\bar{\mu}_{L}+\bar{\mu}_{S}} \right)=\frac{1}{N}\sum_{i=1}^{N} \frac{2(\bar{\mu}_{L}^{i}-\bar{\mu}_{S}^{i})}{\bar{\mu}_{L}^{i}+\bar{\mu}_{S}^{i}},$$

where $\bar{\mu}_{L}$ and $\bar{\mu}_{S}$ refer to within ROI mean LAC in the large and small phantom, respectively. Mean standard deviation $\sigma_{\mu}$ is a within ROI measure of precision and is defined as,

$$\mathrm{mean}\left( \sigma_{\mu} \right)=\frac{1}{N}\sum_{i=1}^{N} \sigma_{\mu}^{i}.$$
